# High-salt in addition to high-fat diet may enhance inflammation and fibrosis in liver steatosis induced by oxidative stress and dyslipidemia in mice

**DOI:** 10.1186/s12944-015-0002-9

**Published:** 2015-02-13

**Authors:** Yuzaburo Uetake, Hitoshi Ikeda, Rie Irie, Kazuaki Tejima, Hiromitsu Matsui, Sayoko Ogura, Hong Wang, ShengYu Mu, Daigoro Hirohama, Katsuyuki Ando, Tatsuya Sawamura, Yutaka Yatomi, Toshiro Fujita, Tatsuo Shimosawa

**Affiliations:** Department of Nephrology and Endocrinology, The University of Tokyo Graduate School of Medicine, Tokyo, Japan; Office for Research Ethics Support, The University of Tokyo Graduate School of Medicine, Tokyo, Japan; Clinical Laboratory, The University of Tokyo Hospital, Tokyo, Japan; Department of Gastroenterology, The University of Tokyo Graduate School of Medicine, Tokyo, Japan; Department of Pathology, Kawasaki Municipal Hospital, Kawasaki, Japan; Department of Gastroenterology, Toshiba General Hospital, Tokyo, Japan; Division of Laboratory Medicine, Department of Pathology and Microbiology, Faculty of Medicine, Nihon University School of Medicine, Tokyo, Japan; Division of Clinical Epigenetics, Research Center for Advanced Science and Technology, The University of Tokyo, Tokyo, Japan; Department of Vascular Physiology, National Cerebral and Cardiovascular Center Research Institute, Osaka, Japan; Department of Physiology, Shinshu University School of Medicine, Nagano, Japan

**Keywords:** High salt diet induced oxidative stress, Oxidative stress, NADPH oxidase, Anti-oxidant tempol, Nonalcoholic fatty liver disease

## Abstract

**Background:**

It is widely known that salt is an accelerating factor for the progression of metabolic syndrome and causes cardiovascular diseases, most likely due to its pro-oxidant properties. We hypothesized that excessive salt intake also facilitates the development of nonalcoholic steatohepatitis (NASH), which is frequently associated with metabolic syndrome.

**Methods:**

We examined the exacerbating effect of high-salt diet on high-fat diet-induced liver injury in a susceptible model to oxidative stress, apoE knockout and lectin-like oxidized low-density lipoprotein receptor-1 (LOX-1) transgenic mice.

**Results:**

High-salt diet led to NASH in high-fat diet-fed LOX-1 transgenic/apoE knockout mice without affecting high-fat diet-induced dyslipidemia or hepatic triglyceride accumulation. Additionally, a high-salt and high-fat diet stimulated oxidative stress production and inflammatory reaction to a greater extent than did a high-fat diet in the liver of LOX-1 transgenic/apoE knockout mice.

**Conclusions:**

We demonstrated that high-salt diet exacerbated NASH in high-fat diet-fed LOX-1 transgenic /apoE knockout mice and that this effect was associated with the stimulation of oxidative and inflammatory processes; this is the first study to suggest the important role of excessive salt intake in the development of NASH.

**Electronic supplementary material:**

The online version of this article (doi:10.1186/s12944-015-0002-9) contains supplementary material, which is available to authorized users.

## Background

Nonalcoholic steatohepatitis (NASH) has been attracting attention recently because of its increasing prevalence and close associations with chronic illnesses, such as diabetes, dyslipidemia and obesity, which are the major components of metabolic syndrome (MetS) [1-6] In addition, several recent studies have reported that there is also a close association between NASH and hypertension [7-9]. Therefore, NASH has been considered to be a hepatic manifestation of MetS. Importantly, the survival rate was reduced in NASH patients, who frequently died from liver-related causes in addition to cardiovascular events [10]. Because NASH is not a benign disease, it is considered to be an important health-threatening component of MetS [11,12].

In the onset and progression of NASH, cholesterol plays a major role in development of NASH causing oxidative stress in hepatic tissue [13]. A high-fat diet (HFD) increases free fatty acids, which induce mitochondrial β oxidation and the overproduction of free radicals [13], both of which play an important role in progression of NASH. In general, patients with NASH tend to consume excessive saturated fatty acid- and cholesterol-enriched foods without supplying anti-oxidative nutrients, such as vitamins E and C; susceptibility to oxidative stress and the excessive intake of lipids may be characteristics of NASH patients [8]. Given the experimental fact that vitamin E and C improved liver damage in NASH [14,15], environmental and intrinsic reactive oxygen species (ROS)-increasing stimuli accelerate the onset and progression of NASH.

Recently several studies have suggested that excessive salt intake would stimulate ROS production in a variety of organs including blood vessels [16], kidneys [17], heart [18] and brain [19] in a model using Dahl salt-sensitive rats. In addition, a high-salt diet (HSD) has also shown to increase oxidative stress in kidneys [20] and arteriolar and venular walls in skeletal muscle in normal rats [21]. Therefore, we hypothesized that excessive salt intake, an oxidative stress-inducer, is an accelerator of the onset and progression of HFD-induced NASH due to ROS overproduction.

To elucidate our hypothesis, we used HFD-fed lectin-like oxidized low-density lipoprotein receptor-1 (LOX-1) transgenic (Tg) and apoE knockout (KO) (TgKO) mice as a model of MetS [22], which follows a chronic course. Because LOX-1, a receptor for oxidized low-density lipoprotein (ox-LDL), which accumulates on the atherosclerotic vasculature, is suggested to cause oxidative stress-related cell damage [23,24], it is expected that LOX-1 overexpression may further augment organ damage by salt-induced ROS overproduction and cause the progression of NASH. In this study, we examined the effect of a high-salt and high-fat diet (HS/HFD) and HFD on liver damage in TgKO mice. In addition, we examined whether the anti-oxidant 4-hydroxy-2, 2, 6, 6-tetramethyl-piperidine-*N*-oxyl (tempol) could ameliorate liver damage in TgKO mice.

## Materials and methods

### Generation of LOX-1 Tg/apoE KO (TgKO) mice

Targeted LOX-1 gene expression in endothelial cells was generated as described previously [25]. Heterozygous LOX-1 Tg mice lines carrying 24 copies of the transgene were crossbred with homozygous C57BL/6:apoE KO mice to generate TgKO were developed as described previously [26] and backcrossed eight times with the C57BL/6 strain to replace the genetic background. Homozygous mutation in the apoE gene was confirmed by Southern blot analysis, as described previously [27].

### Animals and protocols

The present study was conducted in 8-week-old male TgKO mice. They were randomly allocated into groups fed a normal diet (ND) (0.5% salt by weight and 13% fat for calories), HFD (38% fat), or HS/HFD (7.9% salt-containing HFD) for 8 weeks. Some of HS/HFD-fed TgKO mice were treated with an anti-oxidant tempol (Sigma-Aldrich, Mo) (7 mmol/L in drinking water for 8 weeks).

All of the animals received humane care in compliance with the National Research Council’s criteria outlined in the “Guide for the Care and Use of Laboratory Animals” prepared by the US National Academy of Sciences and published by the US National Institutes of Health. The protocol was approved by the Ethics Committee on Animal Research of the University of Tokyo (Permit Number: P08-051). All surgery was performed under sodium pentobarbital anesthesia, and all efforts were made to minimize suffering.

### Liver histology

For histology, 5-μm-thick paraffin sections of liver tissue were prepared from the center of each hepatic lobe. Sections were subsequently stained with hematoxylin and eosin, Masson’s trichrome, or antibodies against 4-hydroxynonenal (4HNE) and F4/80, TNF-α, superoxide dismutase-1 (SOD-1), catalase, glutathione. The disease severity of NASH was assessed according to the grading/staging system of Brunt et al. [28] and the nonalcoholic fatty liver disease (NAFLD) activity score of Kleiner et al. [29].

To examine ROS production, immunohistochemistry of 4HNE (anti-4HNE antibody; JaICA, Shizuoka, Japan) and SOD-1 (anti-SOD-1; sc-11407, Santa Cruz, CA), catalase (anti- catalase; sc-50508, Santa Cruz, CA), glutathione (anti-glutathione; AB-T01, Advanced Targeting Systems, CA), were performed. For the detection of inflammation, the liver sections were stained for F4/80 (anti-F4/80; sc-59171, Santa Cruz, CA) and TNF-α (anti-TNF-a; ab-34674, Abcam, Cambridge, UK).

Histological features of the liver were assessed by two investigators who had no knowledge of the origin of the slides. NAFLD activity score was evaluated on 5 double-blinded random images per animal (n = 6–23) from different lobes of liver.

### Measurement of the biochemical index

The serum total cholesterol level was measured after 8 weeks of diet using ELISA kits (T-Cho H, Wako, Osaka, Japan). Serum alanine transaminase (ALT) activities were measured spectrophotometrically using a commercially available test kit (ALT reagents, Alfresa, Osaka, Japan). Total hepatic triglycerides were extracted from freeze-dried samples by chloroform:methanol (2:1). Triglycerides in hepatic tissue extract were subsequently determined enzymatically in an auto-analyzer using a commercial kit (L type Wako triglycerides H, Wako, Osaka, Japan). As a blood marker for hepatic fibrosis, serum hyaluronic acid (HA) was also assayed using a commercial kit (Lpia ace HA, IATRON, Tokyo, Japan).

### Western blot analysis

Western blotting was performed as described previously [30]. 4HNE, fibronectin and beta-actin were blotted on the same membrane with corresponding monoclonal antibody. Primary antibodies are 4HNE (JaICA, Shizuoka, Japan), fibronectin (sc-71113, Santa Cruz, CA) and β-actin (A-5316, Sigma-Aldrich, St. Louis, MO). Blots were quantified by Scion image analysis.

### Analysis of the mRNA levels of endogenous and transgenic LOX-1

Total RNA was extracted from liver tissues. For Reverse Transcription Polymerase Chain Reaction (RT-PCR) analysis, the cDNA product was synthesized and amplified by PCR using a commercial kit (Thermoscript RT-PCR kit, Invitrogen, CA). Thermal cycler conditions were 35 cycles of 94°C for 40 sec, 60°C for 1 minute, and 72°C for 1 minute for LOX-1.

### Measurement of Nicotinamide Adenine Dinucleotide Phosphate (NADPH) -induced superoxide production in the liver

The production of superoxide anions in the liver was measured using lucigenin chemiluminescence in a microplate luminometer (LB 9507, Berthold Technologies, Germany) as described previously [18].

### Liver perfusion experiments

Mice were anesthetized with pentobarbital (50 mg/kg), and the catheter introduced into the portal vein. Livers were perfused with Krebs-Henseleit bicarbonate buffer saturated with 95% O2 and 5% CO2 at 5 mL/min for 5 minutes at 37°C. Subsequently, livers were perfused with Krebs-Henseleit bicarbonate buffer containing 1 mM of H2O2 for 10 minutes and Krebs-Henseleit bicarbonate buffer containing 0.05% nitro blue tetrazolium (NBT, Sigma-Aldrich, Mo) for 5 minutes. Then, the livers were fixed by 10% formalin, embedded in paraffin, sectioned, and stained with nuclear fast red. The degree of formazan depositions was evaluated by light microscopy [31].

### Statistical analysis

All of the values are expressed as the mean ± SEM. Comparisons among groups were calculated by ANOVA followed by Dunnett’s method, and probability values of *P < 0.05* were considered to indicate statistical significance.

## Results

### RT-PCR analysis of LOX-1 expression in the liver

We confirmed only the expression of LOX-1 transgenes in the liver using RT-PCR analysis. Transgenic bovine LOX-1 and endogenous murine LOX-1 mRNA in the liver was detected (Additional file 1: Figure S1). Endogenous murine LOX-1 and transgenic bovine LOX-1 had comparable signal intensity for an equal molar level of LOX-1, as previously reported [24].

### Systolic blood pressure

Systolic blood pressure was elevated in both HFD and HS/HFD groups to the same extent (114.2 ± 0.3 mmHg and 118.4 ± 3.3 mmHg; *P < 0.05, P < 0.05* compared with ND: 106.1 ± 1.0 mmHg, respectively).

### Serum lipids

The serum level of total cholesterol in mice fed ND was elevated (556 ± 34 mg/dL). Both HFD and HS/HFD significantly increased the serum level of total cholesterol (1042 ± 53 mg/dL and 1239 ± 111 mg/dL; *P < 0.001, P < 0.001* compared with ND, respectively). The cholesterol level between HFD and HS/HFD group were comparable (*P = 0.10*). Thus, salt intake did not affect cholesterol metabolism.

### Fat accumulation in the liver and serum alanine transaminase

**Figure 1:**
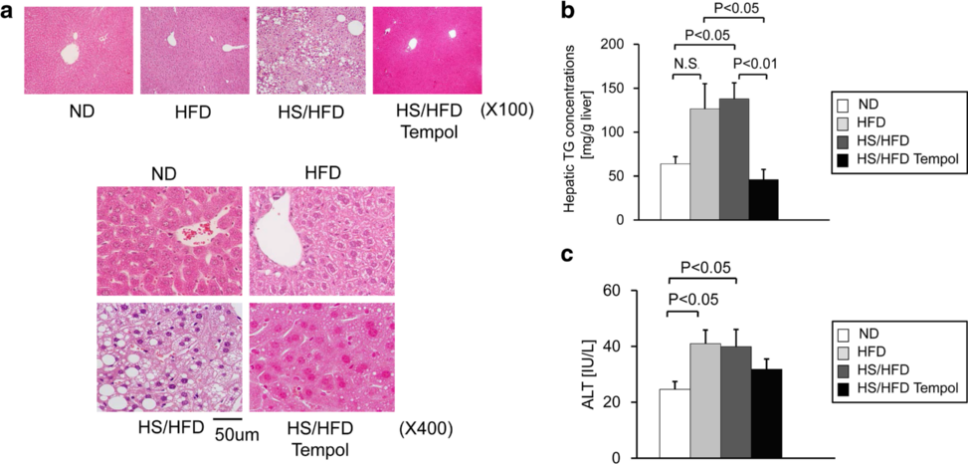
**Fat accumulation in the liver and liver function in mice with different dietary treatments. a**, Representative hepatic histological findings (HE staining: original magnification, X100, X400). **b**, The effects of normal diet (ND), high-salt and high-fat diet (HS/HFD), high-fat diet (HFD) and HS/HFD feeding plus 4-hydroxy-2,2,6,6-tetramethyl-piperidine-*N*-oxyl (tempol) treatment (HS/HFD Tempol) on hepatic triglyceride concentrations (n = 4-14/group). **c**, Serum alanine transaminase (ALT) activities (n = 5-11/group). Values are means ± SEM.

**Figure 2:**
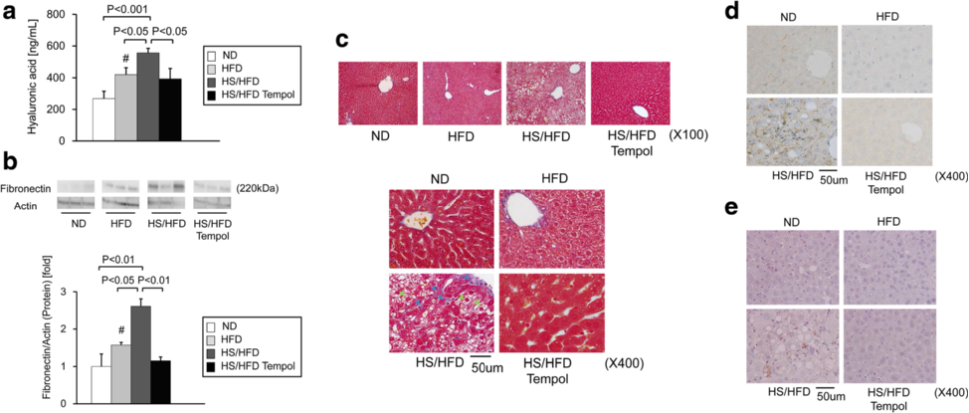
**Evaluation of fibrosis and inflammation in the liver of mice with different dietary treatments. a**, Serum hyaluronic acid levels. (n = 6-14/group) **b**, Fibronectin protein levels in the whole liver (n = 4/group). **c**, Histological analysis of fibrotic changes. Representative fibrilar collagen deposition in the liver, which was evaluated using Masson’s trichrome staining. Fibrotic changes are indicated by blue arrowheads and typical hepatocyte ballooning are indicated by green arrowheads (original magnification, X100, X400). **d**, Histological analysis of inflammatory changes. Representative F4/80 immunohistochemical stainning (original magnification, X400). **e**, Histological analysis of inflammatory changes. Representative TNF-α immunohistochemical stainning (original magnification, X400). See abbreviations in the legends of Figure 1. ^*#*^ *P < 0.05* compared with mice fed ND. Values are means ± SEM.

**Figure 3:**
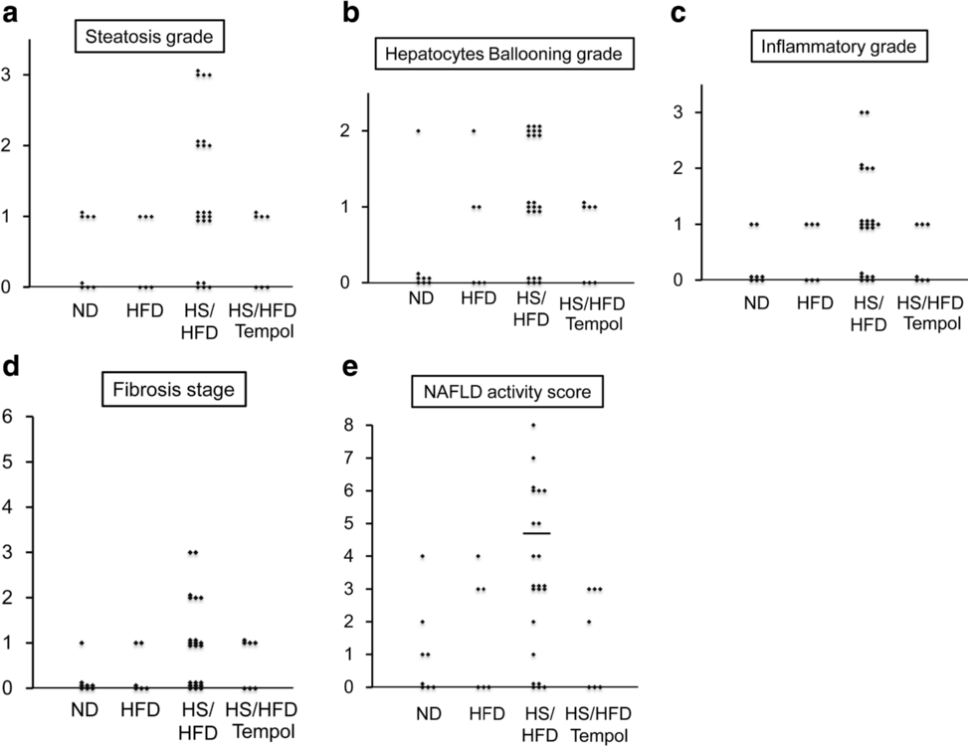
**Evaluation of NASH by NAFLD activity score. a**, steatosis grade. **b**, hepatocyte ballooning grade, and **c**, inflammatory grade. **d**, fibrosis stage. **e**, NAFLD activity score. Five points or more of NAFLD activity score were evaluated for diagnosis of NASH. Solid lines indicate the border of five points. n = 6-23/group. See abbreviations in the legends of Figure 1.

Similar to serum cholesterol level, hepatic triglyceride (TG) concentrations were comparable between HFD and HS/HFD groups (Figure 1b). In comparison with ND group, HS/HFD showed statistically significant difference *(P < 0.05)* and HFD tended to increase hepatic TG concentrations (Figure 1b). It is because the hepatic TG concentrations varies sample to sample in HFD group (Figure 1a and b) and statistically there is tendency in higher hepatic TG concentrations compare with ND group. Moreover, the degree of steatosis did not correspond with hepatic TG concentrations in HFD group (Figure 1a and b and Figure 2c and Figure 3a), which was similar to the former studies [32,33]. ALT level was higher both in HFD and HS/HFD groups than ND and there were no additive effect of salt intake *(P < 0.05, P < 0.05*; Figure 1c).

### Serum HA and fibronectin expression in the liver

To examine the progression of fibrosis in steatohepatitis, we evaluated serum HA values (Figure 2a). Salt loading increased HA. Similar with HA, salt loading increased fibronectin expression in the liver of HS/HFD group (2.6-fold) compared to ND group (P < 0.05; Figure 2b).

### Evaluation of NASH

Macrovesicular steatosis and pericellular fibrosis in the liver were only found in mice fed HS/HFD but was not apparent in the groups fed ND and HFD (Figure 1a and Figure 2c). It is noteworthy that the most apparent changes for steatosis were found in HS/HFD group (Figure 1a and Figure 2c and Figure 3a). Fibrosis was localized around the central vein and throughout the lobule in a pericellular distribution. This alteration in mice was compatible to the diagnostic criteria of human NASH. The ballooning of hepatocytes was found in mice fed HS/HFD (Figure 1a and Figure 2c and Figure 3b). HFD group showed a slight tendency of the ballooning of hepatocytes, suggesting that excess salt contributes to the damaging process of the liver. Fibrotic changes in the liver were apparent only in HS/HFD group (Figure 1a and Figure 2c and Figure 3d).

We examined inflammation in the liver by evaluating inflammatory cell recruitment and immunostaining F4/80 and TNF-α (Figure 2d and e). The liver of HS/HFD group exhibited active inflammation, and salt aggravated inflammation in comparison with HFD group (Figure 1a and Figure 2c, d and e and Figure 3c). Additionally, NASH assessment was performed on all applicable mice. As a result, we diagnosed NASH in eight mice from HS/HFD mice group (n = 23) (35%) (Figure 3e). No other mice from other groups had NASH.

### Accumulation of Hepatic ROS in NASH

**Figure 4:**
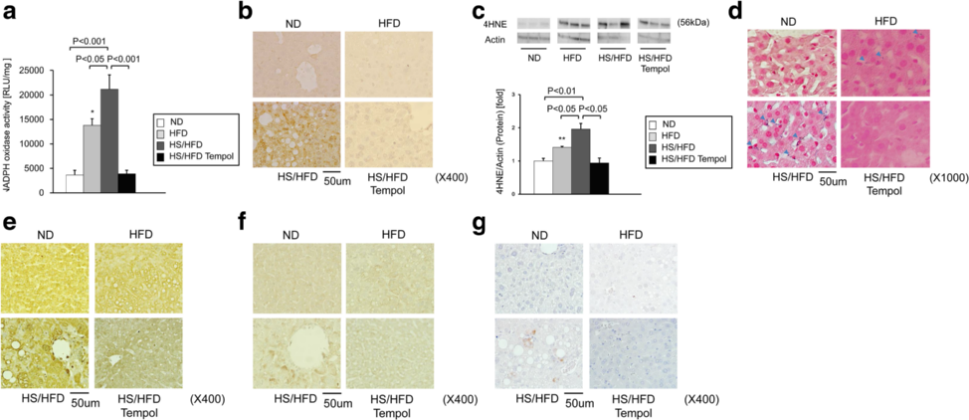
**Hepatic ROS accumulation in the liver of each treated group of mice. a**, The NADPH oxidase activity (n = 6-11/group). **b**, Representative histological findings with immunohistochemical staining for 4-hydroxynonenal (4-HNE: original magnification, X400). **c**, 4-HNE protein in the whole liver (n = 4 for each group). **d**, Oxygen radical formation in Kupffer cells after H2O2 preconditioning. Formazan depositions are indicated by blue arrowheads. (nuclear red staining: original magnification, X1000). **e**, Histological analysis of anti-oxidant enzymes activity. Representative SOD-1 immunohistochemical stainning (original magnification, X400). **f**, Histological analysis of anti-oxidant enzymes activity. Representative catalase immunohistochemical stainning (original magnification, X400). **g**, Histological analysis of anti-oxidants. Representative glutathione immunohistochemical stainning (original magnification, X400). See abbreviations in the legends of Figure 1. Values are means ± SEM. *P < 0.001 compared with mice fed ND. **P < 0.01 compared with mice fed ND.

NADPH oxidase activity in the liver was measured by the lucigenin chemiluminescence method (Figure 4a). HFD stimulated NADPH oxidase activity (*P < 0.05*) and salt aggravated NADPH oxidase activity (*P < 0.001*).

Next, to confirm the accumulation of oxidative stress, we performed immunostaining of 4HNE–adducted protein in liver sections. Lipid peroxidation was markedly elevated in HS/HFD group compared with ND or HFD groups (Figure 4b). We quantified the amount of lipid peroxidation by Western blot analysis of 4HNE in the liver. 4HNE was increased by approximately 2.0-fold in HS/HFD (*P < 0.01* compared with ND, respectively), but only a small increase in 4HNE was observed in HFD group. Salt loading further increased 4HNE expression significantly (*P < 0.05* compared with HFD, respectively; Figure 4c).

Moreover, we performed immunostaining of SOD-1, catalase, glutathione in liver sections to confirm anti-oxidant enzyme and anti-oxidants (Figure 4e, f and g). The liver of HS/HFD group exhibited diffuse staining of SOD and catalase in comparison with HFD group. Otherwise, we could confirm staining only the liver of HS/HFD group of glutathione.

### Production of ROS in sinusoidal cells after H2O2 preconditioning

To elucidate the contribution of sinusoidal cells, we determined the amount of ROS produced in sinusoidal cells with H2O2 preconditioning. As a result, H2O2 preconditioning only induced formazan depositions in Kupffer cells from HS/HFD or HFD group (Figure 4d). Salt loading showed an additive effect on formazan depositions.

### Effects of anti-oxidant, tempol, on NASH in HS/HFD-fed mice

The present findings suggest that excessive salt intake could be accelerated the onset and progression of HFD-induced NASH due to ROS overproduction. To clarify this hypothesis, we examined the effects of tempol on liver damage in HS/HFD-fed mice. Tempol did not improve total cholesterol levels in HS/HFD-fed mice (1020 ± 135 mg/dL). However, the hepatic accumulation of lipid droplets and triglyceride concentration were normalized by tempol (Figure 1a and b). Additionally, tempol restored serum HA values (Figure 2a) and hepatic fibronectin expression (Figure 2b). Tempol also ameliorated steatosis, hepatocyte ballooning, fibrosis, and inflammation in the liver (Figure 3a, b, c and d). As a result, NASH was not found in tempol-treated HS/HFD-fed mice (Figure 3e). Antioxidant effects of tempol were confirmed by the measurement of NADPH oxidase activity (Figure 4a) and 4HNE expression (Figure 4b and c) in the liver and oxygen radical formation in Kupffer cells after H2O2 preconditioning (Figure 4d).

## Discussion

This study first confirms that salt loading on HFD may accelerate induction of NASH. HS/HFD induced significant fatty degeneration around the hepatic central veins associated with fibrotic changes. The deterioration of fatty metabolism, inflammation, and pericellular fiborosis observed in HS/HFD-fed mice met the criteria of NASH in human [28,29], and Kleiner scores were significantly higher in HS/HFD-fed mice (Figure 3) [29]. These fibrotic changes were comparable with serum HA level and fibronectin expression in the liver both of which were significantly elevated in HS/HFD-fed mice.

In the current model, salt loading accelerated the onset and progression of NASH in 8 of 23 mice (35%) in a short term (8 weeks) (Figure 3e). In the pathological criteria of human NASH, in addition to fatty degeneration and fibrosis, pericellular hepatocyte ballooning is regarded as a crucial pathologic finding in diagnosis of NASH in human. Interestingly, the current study confirmed that hepatocyte ballooning occurred earlier than fatty degeneration in several samples when salt loading was added in HFD-fed mice. In this group with salt-overload, histopathologic inflammatory and fibrotic changes were more remarkable and serum HA and fibronectin levels were significantly higher (Figure 2a and b) compared to HFD-fed mice without salt-overload.

Meanwhile, the question is that how salt loading can affect the induction of NASH. Our results suggested that the level of oxidative stress was exaggerated when salt intake was overloaded in HFD-fed mice (Figure 4a, b, c and d). Systolic blood pressure was elevated in both HFD and HS/HFD groups to the same extent, but we confirmed NASH only in HS/HFD group. Therefore, the present findings suggest that excessive salt intake could accelerate the onset and progression of ROS-induced NASH. Hypertension may not be essential to the development of NASH. In this study, we measured oxidative stress by 4HNE and confirmed its origin by liver perfusion experiments (*ex vivo*) using NBT (Figure 4d). Our results suggested that Kupffer cells and sinusoidal endothelial cells seemed to produce oxidative stress both in HS/HFD-fed mice and HFD-fed mice (Figure 4d). Serum concentrations of lipids were significantly increased both in HS/HFD- and HFD- mice, and these observations were compatible with well-known hypothesis that cholesterol enhances ROS production [13]. However, the interesting observation in this study was that salt overload further stimulated ROS production in HS/HFD-fed mice compared to HFD-fed mice. This result suggests that salt overload may also induce ROS production and exacerbate ROS-associated damage in liver tissue. Moreover, we have performed immunostaining for SOD and catalase, anti-oxidant enzyme, and glutathione (anti-oxidants) to confirm the activity of anti-oxidant enzyme and anti-oxidants. The liver of HS/HFD group exhibited diffuse staining of SOD and catalase in comparison with HFD group. Otherwise, we could confirm staining only the liver of HS/HFD group of glutathione. In the HS/HFD group, even glutathione were produced, glutathione could not be erased ROS, and then NASH was developed. These results suggest that in HS/HFD groups, ROS was not fully erased by glutathione and residual oxidative stress contributed to induction of NASH. However, correlation of lipid metabolism and salt intake is not clear.

Another noteworthy result was that tempol, a SOD mimetic, could reduce the NASH-like histopathologic changes in the liver in HS/HFD-fed mice. Tempol scavenges ROS by NADPH oxidase. It has been reported that ROS itself can activate NADPH oxidase probably in the feed-forward loop in the vascular system [34-36], and in turn, reduction of ROS with a radical scavenger, tempol, can interrupt NADPH oxidase activity. In the present study, tempol could restore both NADPH oxidase activity (Figure 4a) and ROS as demonstrated by 4HNE expression (Figure 4b and c) in the liver and oxygen radical formation in Kupffer cells after H2O2 preconditioning (Figure 4d). Because tempol scavenges only ROS and would not directly affect the activity of NADPH oxidase, the feed-forward activation mechanism between NADPH and ROS might also present in the liver [18].

The limitations of this study include that the model used in this study was highly susceptible to fat-overload and salt loading was relatively strong. Thus, it remains difficult to determine how much salt loading increase the risk of NASH. In addition, the detailed mechanism of ROS production in salt loading is still unclear, and further investigation is needed. However, the current results clearly indicated the possibility that salt overload may accelerate induction of NASH in mice susceptible to HFD. Furthermore, our model can offer a better model of NASH compared to conventional animal models requiring longer-term dietary modifications or several genetic engineering. This model is characterized with diet-induced overproduction of oxidative stress and genetic backgrounds for MetS. Therefore, it could be used to evaluate the effects both environmental and genetic predisposition for NASH.

In conclusion, salt overload may accelerate the accumulation of oxidative stress under the condition of HFD consumption and induce NASH in the liver in a dyslipidemia model, LOX-1 Tg and apoE KO mice. Although further investigation is needed to clarify how much salt intake affects the ROS production and progression of chronic liver damage, this study would trigger the experimental interest whether or not salt intake is important in progression of liver disease.

## Additional file

Additional file 1: Figure S1. Lectin-like oxidized LDL receptor-1 (LOX-1) expression. Transgenic bovine lectin-like oxidized LDL receptor-1 (LOX-1) and endogenous murine LOX-1 mRNA measured by reverse transcription polymerase chain reaction (RT-PCR).

## Abbreviations

MetS: Metabolic syndrome
NAFLD: Nonalcoholic fatty liver disease
NASH: Nonalcoholic steatohepatitis
ND: Normal diet
HSD: High-salt diet
HFD: High-fat diet
HS/HFD: High-salt and high-fat diet
LOX-1: Lectin-like oxidized low-density lipoprotein receptor-1
Tg: Transgenic
TgKO: LOX-1 transgenic/apoE KO
ROS: Reactive oxygen species
ox-LDL: Oxidized low-density lipoprotein
tempol: 4-hydroxy-2,2,6,6-tetramethyl-piperidine-*N*-oxyl
4-HNE: 4-hydroxynonenal
ALT: Alanine transaminase
HA: Hyaluronic acid
RT-PCR: Reverse Transcription Polymerase Chain Reaction
NADPH: Nicotinamide Adenine Dinucleotide Phosphate
NBT: Nitro blue tetrazolium
SOD: Superoxide dismutase
