# Supplementary figures and images for: High-salt in addition to high-fat diet may enhance inflammation and fibrosis in liver steatosis induced by oxidative stress and dyslipidemia in mice
Source: Lipids Health Dis. 2015 Feb 13;14:6. doi: 10.1186/s12944-015-0002-9 (PMC4337194; doi:10.1186/s12944-015-0002-9)

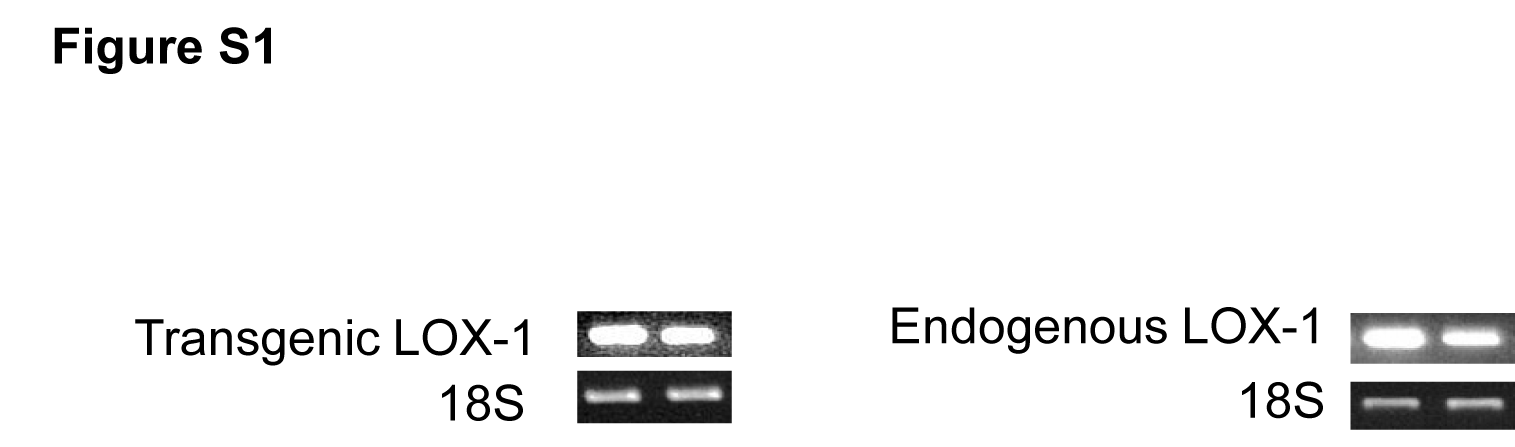

Supplement: Additional file 1: Figure S1. — Lectin-like oxidized LDL receptor-1 (LOX-1) expression. Transgenic bovine lectin-like oxidized LDL receptor-1 (LOX-1) and endogenous murine LOX-1 mRNA measured by reverse transcription polymerase chain reaction (RT-PCR). [file 12944_2015_2_MOESM1_ESM.tiff]
